# Seminal glucose levels: a prognostic factor of sperm survival to cryopreservation?

**DOI:** 10.5935/1518-0557.20230049

**Published:** 2023

**Authors:** Séphora C. Queiroz, Maíra Casalechi, Simone F. Nery, Cynthia Dela Cruz, Augusto B. Reis, Fernando M Reis

**Affiliations:** 1 Department of Obstetrics and Gynecology Universidade Federal de Minas Gerais, Belo Horizonte, Brazil; 2 Department of Surgery, Universidade Federal de Minas Gerais, Belo Horizonte, Brazil

**Keywords:** semen, glucose, cryopreservation, infertility

## Abstract

**Objective:**

Considering that glucose is an important component of seminal plasma and is a cryoprotectant at high concentrations, the aim of this study was to investigate the possible association of glucose levels in fresh semen with the sperm survival and motility rates following cryopreservation.

**Methods:**

This was a prospective study including 149 men undergoing semen analysis due to male and/or female infertility. The seminal samples were analyzed according to the World Health Organization standards and glucose concentrations were measured using a dipstick glucometer. Samples were cryopreserved with Test Yolk Buffer-Gentamicine freezing medium under liquid nitrogen for an average of 120 days. The frozen aliquots were thawed at 37°C for 10 minutes and analyzed using the same methods and protocols used pre-freezing.

**Results:**

Glucose levels ranged from 14 to 99 mg/dL and were similar in individuals with normal (n=100) *vs*. abnormal (n=49) semen analysis. The rates of sperm recovery (total, alive or motile sperm) in the cryopreserved samples did not change among samples with different glucose levels (*p*>0.05, Kruskal-Wallis ANOVA and Spearman’s correlation coefficient).

**Conclusions:**

There appears to be no association between glucose levels in human semen samples and their resistance to cryopreservation.

## INTRODUCTION

Approximately one in ten adult men is infertile, and half of them seek medical help to conceive (Datta *et al*., 2016). Male factor infertility is defined as infertility caused primarily by abnormal semen parameters or function, abnormalities of the male reproductive system, or inadequate sexual function and ejaculation (Zegers-Hochschild *et al*., 2017). When spermatogenesis is so severely compromised that no sperm can be recovered in the ejaculate or even through testicular biopsy, the couple may decide to use heterologous semen obtained from gamete banks. The availability of donor sperm presupposes cryopreservation, so samples can be safely processed, screened for infectious agents, stored, and shipped. Sperm cryopreservation is also useful for fertility preservation in men undergoing medical or surgical interventions that are potentially sterilizing (Practice Committee of the American Society for Reproductive Medicine & Practice Committee for the Society for Assisted Reproductive Technology, 2021).

Some substances found in the seminal fluid have an important role in the preservation of frozen semen (Muiño-Blanco *et al*., 2008; Colás *et al*., 2009; Queiroz *et al*., 2020). Glucose plays a significant role in sperm motility and is a required substrate to support acrosome reaction during the capacitation process (Williams & Ford, 2001; Marín-Briggiler *et al*., 2021). The availability of glucose is essential to maintain human sperm motility in vitro during 24h and keep their full potential to fertilize oocytes (Mahadevan *et al*., 1997). The addition of exogenous glucose solution during in vitro incubation increases sperm motility, mitochondrial function and vitality for up to 10 days at room temperature or four days at 37°C (Amaral *et al*., 2011). However, it is still unknown whether endogenous glucose levels in fresh semen samples correlate with their survival and viability after cryopreservation.

Thus, the aim of this study was to investigate the possible association of glucose levels in fresh, undiluted human semen with the sperm survival and motility rates following cryopreservation.

## MATERIAL AND METHODS

### Patients, samples and semen analysis

Seminal samples were obtained from 149 consecutive men attending the reproductive medicine unit of an academic hospital in Belo Horizonte, Brazil, for diagnosis and treatment of infertility. The study protocol was approved by the Research Ethics Committee of Universidade Federal de Minas Gerais and all participants provided written informed consent.

Each patient donated only one sample for the study. Samples were collected by masturbation into non-toxic sterile collectors and maintained at 37ºC on a warm plate until complete liquefaction. Seminal samples were analyzed within 60 minutes after ejaculation for morphology, concentration, motility and vitality (Vieira *et al*., 2012) using established standard criteria according to WHO guidelines (Cooper *et al*., 2010). The study population was divided according to the results of the seminal analysis as normal (n=100), defined as having sperm concentration ≥15million/ml, total sperm number ≥39million and progressive motility ≥32% according to the updated WHO reference limits; or abnormal (n=49) when at least one seminal parameter was below the reference values.

Azoospermic patients were excluded, as well as those who had sperm morphology with normal forms < 4%, semen volume <2ml, progressive motility = 0%, or total sperm count < 1 million per ejaculate. These criteria were chosen to avoid post-thawing motile sperm counts below the detection limit of the analytic method.

### Glucose Quantification

Glucose quantification was performed using test-strips Accu-Check Active^®^, Roche^®^. Briefly, a drop of fresh semen was placed on a specific area of the dipstick and read immediately in a glucometer. The results provided by the glucometer were expressed as mg/dL, ordered and classified into tertiles. The 1^st^ tertile had glucose values between 14 and 31 mg/dL; the 2^nd^ tertile was between 32 and 45 mg/dL, and the 3^rd^ tertile was between 46 and 99 mg/dL.

### Cryopreservation

The time between sample collection and freezing ranged from 30 minutes to 1 hour. Semen freezing was performed using a rapid freezing protocol (Vieira *et al*., 2012; Queiroz *et al*., 2020). Test Yolk Buffer with Gentamicine (TyB-G) and 12% Glycerol freezing medium (Irvine Scientific, Santa Ana, CA, USA) previously stored at -20°C were thawed at room temperature for 30 minutes and added dropwise to cryopreservation tubes containing the fresh semen samples in a 1:1 ratio for a total volume of 1 ml.

The cryotubes were fixed in racks positioned horizontally on the vapor of liquid nitrogen in a polystyrene foam box, 10 cm above the liquid surface for 10 minutes, and then were plunged into liquid nitrogen and stored for a median period of 120 days (interquartile interval 91-122 days). Then, the frozen aliquots were thawed at 37°C for 10 minutes and analyzed using the same methods and protocols used before freezing. All analyses were performed blind to patient identification and to the baseline semen parameters.

### Statistical analysis

Since most variables had non-normal distribution, they were summarized as medians and interquartile intervals. Differences between groups were analyzed with Brunner-Munzel test (two groups) (Karch, 2021) or Kruskal-Wallis ANOVA followed by Dunn’s test for multiple comparisons (three groups). Spearman’s rank correlation coefficients were calculated to test the association between glucose levels and other quantitative semen parameters in fresh and frozen samples. The sample size was calculated to detect differences of at least 20% in the sperm recovery rates between groups with different seminal glucose levels, with alpha = 0.05 and statistical power = 0.8.

## RESULTS

Apart from the parameters used to define the two groups (sperm count, concentration, and motility) the groups with normal and altered seminal analysis differed only by age (median 37 *vs*. 35 years, Table 1). There was no difference between the groups regarding the time of abstinence, sample volume, or concentration of seminal glucose (Table 1).

**Table 1:** Characteristics of the patients and their seminal analyses before cryopreservation.

	Normal (n=100)	Altered (n=49)
Age (years)	37 (33-40)	35 (31-37)^*^
Abstinence (days)	5 (4-5)	5 (4-5)
Semen volume (ml)	3.3 (2.5-4.4)	3.0 (1.9-4.0)
Total sperm number (x106 per ejaculate)	189 (119-425)	26 (13-70)^*^
Sperm concentration (x106 per ml)	80 (35-122)	9 (5-57)^*^
Total motility (%)	70 (60-75)	55 (50-68)^*^
Progressive motility (%)	60 (25-90)	40 (0-60)^*^
Vitality (%)	89 (86-92)	86 (80-92)
Glucose (mg/dl)	41 (30-48)	36 (27-56)

Data are presented as medians (interquartile intervals).

* *p*<0.05, Brunner-Munzel test.

**Figure 1:**
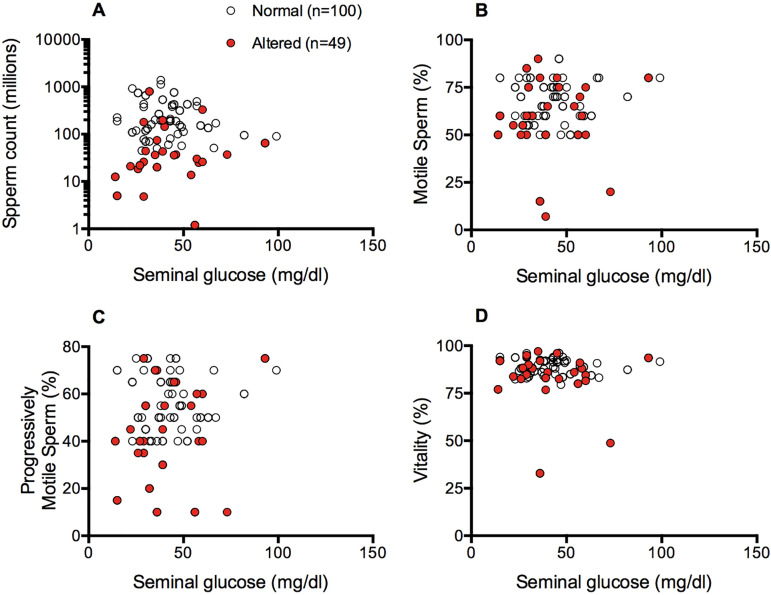
Linear correlation analyses between glucose levels and semen parameters in fresh samples.

In fresh samples, seminal glucose levels correlated positively but weakly with sperm count only in the group with altered semen analysis (Sperman’s r=0.301, n=49, *p*=0.04, Figure 1A). However, glucose levels did not correlate with sperm motility or vitality in any of the groups (Figure 1 B-D).

**Figure 2:**
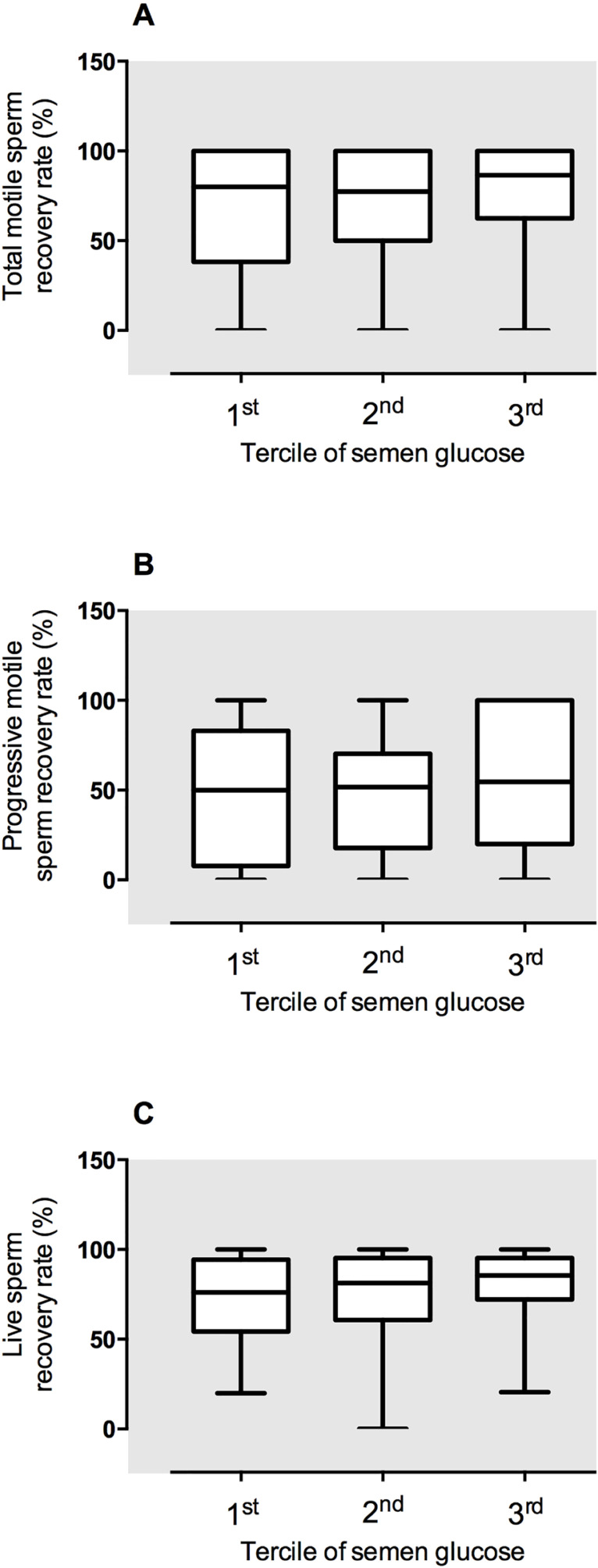
Post-thawing outcomes of all seminal samples (n=149) according to their glucose concentrations measured with dipstick before cryopreservation.

**Figure 3:**
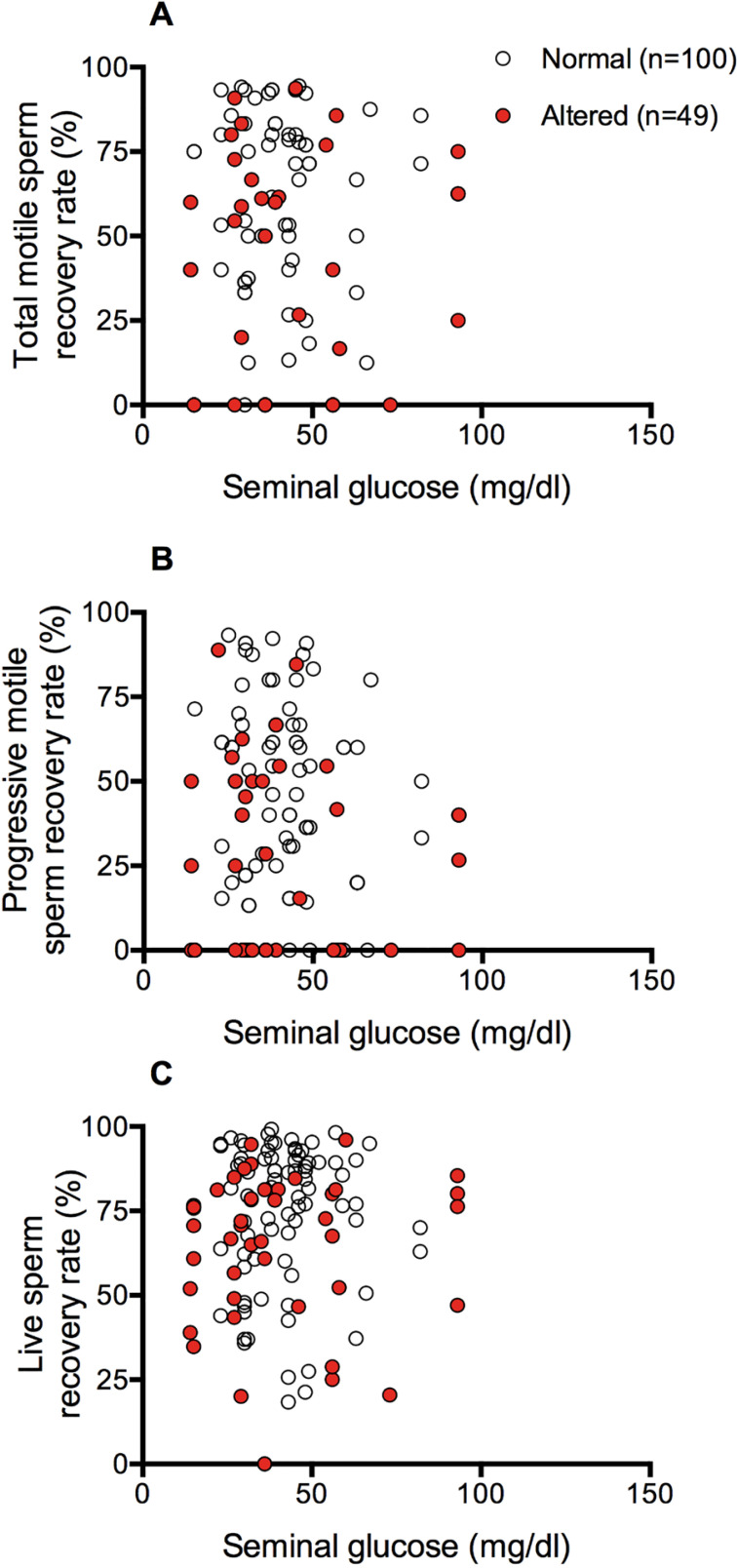
Linear correlation analyses between glucose levels in fresh samples and sperm recovery after cryopreservation.

When we analyzed the possible association between glucose levels in fresh samples and their resistance to cryopreservation, we observed that the sperm recovery rates were similar between samples with different glucose concentrations (*p*>0.05, Kruskal-Wallis ANOVA, Figure 2). This was confirmed by the linear correlation analysis that showed no correlation between glucose levels in fresh samples and sperm recovery after cryopreservation (Figure 3).

## DISCUSSION

In the present study, we investigated the possible relationship between glucose levels in human semen and its resistance to cryopreservation. Our hypothesis was that semen samples with higher glucose levels would be more protected from cryoinjury, considering that glucose protects sperm from low temperature damage (McGonagle *et al*., 2002) and prolongs sperm vitality, motility and fertilizing potential in vitro (Mahadevan *et al*., 1997; Amaral *et al*., 2011). However, we found no evidence of association between endogenous glucose levels and cryopreservation outcomes in human semen samples. Exogenous nutrients, including glucose, are critically needed to keep sperm alive and preserve their progressive motility as well as their capacitation, i.e., the metabolic and kinetic changes that render the sperm capable of penetrating the zona pellucida to fertilize the oocyte (Marín-Briggiler *et al*., 2021). During cryopreservation, the cell enters a state of minimum metabolism (Whaley *et al*., 2021) and exogenous energy sources may no longer be required, so the availability of glucose in the seminal plasma loses importance once the cell temperature is stabilized at very low level. On the other hand, the sperm survival to cryopreservation is affected by protective factors like antioxidants that are present before freezing and attenuate cell damage henceforth (Shokri *et al*., 2019).

In fresh semen, there was a weak positive correlation between seminal glucose and total sperm count, but not between glucose levels and sperm motility or vitality. Only one previous study measured glucose in fresh human semen and found no difference between normal, oligospermic, azoospermic and vasectomized men (Diamandis *et al*., 1999), corroborating with our present findings. As far as we know, the present study is the first to analyze seminal glucose concentrations before cryopreservation. We found no association between glucose levels and the rates of sperm recovery (total, alive or motile sperm) in the cryopreserved samples. It is well established that high concentrations of glucose in the cryoprotectant solution prevent cryodamage and increase the recovery of motile sperm (McGonagle *et al*., 2002; Bhat *et al.*, 2020). Therefore, our findings suggest that this protective effect does not change with the physiological variations of the amount of endogenous glucose that is present in fresh semen, but only with the supraphysiological levels attained by the addition of exogenous glucose.

A strength of this study is the hypothesis-driven, prospective design, with all samples handled equally and blindly, a simple and accurate method of glucose measurement and a statistically robust sample of men with normal as well as altered semen. Some limitations, however, should be noted. We only investigated individuals referred to the clinic for couple infertility, therefore our data should not be automatically extrapolated to typical semen donors, to oncological patients or to transgender females seeking fertility preservation. This limitation was due to the ethical decision to perform the study using surplus samples after routine semen analysis instead of samples from patients requiring cryopreservation.

In conclusion, there appears to be no association between glucose levels in human semen samples and their resistance to cryopreservation. While glucose is an important component of seminal plasma and is a cryoprotectant at high concentrations, the measurement of endogenous glucose levels does not help to predict the sperm recovery rate after cryopreservation of human semen.
